# Broken Rotor Bar Fault Detection for Inverter-Fed Induction Motor with Negative-Sequence Current Analysis

**DOI:** 10.3390/s25227045

**Published:** 2025-11-18

**Authors:** Sarvarbek Ruzimov, Jianzhong Zhang, Xu Huang, Muhammad Shahzad Aziz

**Affiliations:** School of Electrical Engineering, Southeast University, Nanjing 210096, China; s.k.ruzimov@seu.edu.cn (S.R.); huangxuee@seu.edu.cn (X.H.); shahzadaziz@seu.edu.cn (M.S.A.)

**Keywords:** broken rotor bar, induction motor, fault diagnosis, negative-sequence current, Kalman filter, condition monitoring

## Abstract

This study examines the effectiveness of negative-sequence current analysis in identifying broken rotor bars for inverter-fed induction motors under different load and speed conditions. To improve diagnostic reliability, inverter-induced harmonics in the negative-sequence current were separated using a Spectrum-Tracking Hybrid Adaptive Extended Kalman Filter method, resulting in a clearer signal representation. A thorough assessment of the fault detection accuracy and sensitivity was performed to measure fault visibility and clarity. Visual comparisons of healthy and faulty negative-sequence current signals validated comparable patterns of anomalies. The findings validate that negative-sequence current analysis, enhanced by extended Kalman filter-based harmonic isolation, is a dependable and resilient technique for detecting broken rotor bars, especially in the early stages of faults and under various operating conditions.

## 1. Introduction

Induction motors (IMs) are used in industrial applications because of their longevity, simplicity, and efficiency [1,2]. Despite their benefits, they can shatter rotor bars, which is a major issue referred to as a broken rotor bar (BRB) fault, which can result in torque instability, elevated vibration levels, overheating, and, in severe cases, total motor failure [3]. The BRB fault can reduce motor performance and cause costly downtimes if it is not caught early.

Different types of faults are known to introduce specific frequency components into current spectra, which can be effectively used for fault identification and severity assessment [3]. The harmonic components present in the signal spectrum of electrical machines originate mainly from three sources: (i) the machine, (ii) the power supply system, and (iii) any existing faults. The harmonics produced by the machine are affected by factors including non-sinusoidal winding distribution, variations in air-gap permeance resulting from mechanical imperfections, and material nonlinearity. Moreover, anisotropies in the stator and rotor, such as slot openings and minor eccentricities in the air gap during normal operating conditions, lead to the phenomenon referred to as principal slot harmonics or rotor slot harmonics [4]. Power supply-related harmonics are generally minimal in grid-fed machines; however, they become considerably more pronounced in inverter-fed systems due to the inherent switching behavior of power electronics. The amplitude and frequency of harmonics in inverter-fed induction motors are primarily influenced by the inverter’s switching frequency and the modulation technique utilized in its control algorithm. The switching frequency of power electronic devices involves a trade-off between conversion efficiency and the level of low-order harmonics. Increasing the switching frequency reduces low-order harmonics; however, it concurrently elevates switching losses in the inverter. A BRB in an IM generally results from structural or material faults in the rotor, or from thermal and mechanical loads encountered during operation [5]. In healthy motors functioning under steady-state circumstances, the stator current spectrum ideally comprises solely the supply frequency component. When a rotor bar is broken, the rotor exhibits electrical and magnetic asymmetry, resulting in the introduction of supplementary frequency components into the air-gap flux. Sideband harmonics manifest at the frequency (1 ± 2*s*)*f_s_*, where *s* denotes the slip. The harmonics manifest in the stator current, and as the process progresses, they generate further harmonic families at (1 ± 2k*s*)*f_s_*, resulting in a series of fault-related sidebands. The characteristic frequencies related to BRB can be articulated as *f*_brb_ = (1 ± 2k*s*)*f_s_*. Consequently, the majority of diagnostic methods for BRB concentrate on identifying and examining the sideband frequencies within the stator current spectrum, as their existence and magnitude serve as significant markers of rotor asymmetry and bar failure [5]. However, the frequencies of the lower and upper sidebands (LSB and USB) related to the BRB fault are situated near the fundamental frequency. Consequently, these low-order harmonics may obscure the fault-related components and induce spectral leakage, which can result in misdiagnosis when using conventional algorithms.

Condition-monitoring techniques for identifying faults in an IMs can be categorized into invasive and non-invasive methods [6]. Invasive techniques, such as vibration analysis [7], magnetic flux monitoring [8], necessitate the installation of specialized sensors in the motor components, rendering them more complex and expensive. In contrast, non-invasive approaches utilize externally obtainable signals without modifying the motor structure, rendering them more straightforward and easier to employ. This includes the monitoring of stator current, rotor speed [9,10], electromagnetic torque, auditory emissions, and instantaneous power. Some approaches, such as auditory and torque measurements, necessitate the installation of external sensors on the motor body, but others, like motor current signature analysis (MCSA), can be conducted solely through electrical measurements at the supply terminals [11]. The majority of these condition-monitoring approaches are carried out online, continuously monitoring fault-related signals while the machine operates. This classification emphasizes the trade-off between precision and practicality in fault detection, where invasive techniques provide great sensitivity but are less convenient, whilst non-invasive approaches are more suitable for industrial implementation [12].

Early-stage BRB faults have weak signal effects, which makes such flaws hard to detect. Overcoming this, ref. [13] proposed a hybrid diagnostic framework combining Electrical Time-Synchronous Averaging (ETSA), Discrete Wavelet Transform (DWT), and Fuzzy Logic Algorithm (FLA), which proved effective in isolating fault-specific signal components and distinguishing between healthy and degraded rotor conditions with high sensitivity. Researchers have developed sophisticated signal-processing methods to improve fault detection under noisy or low-load conditions in conventional MCSA. By combining the Normalized Least Mean Square (NLMS) filter and Hilbert envelope analysis, it can be seen that these improve the SNR and make it easier to detect the BRB faults [14]. Wavelet Packet Decomposition (WPD) has been used to recover transient and non-stationary signal characteristics from rotor anomalies, improving sensitivity and resolution over Fourier-based methods [15]. The signal-processing methodologies, such as Hilbert transforms and wavelet-based demodulation, have shown potential in the detection of low-severity faults with improved sensitivity [16].

In recent years, machine learning (ML) and deep learning (DL) have been employed to improve automation and diagnostic accuracy. For instance, convolutional neural networks (CNNs) are used to categorize greyscale current signal representations and achieve fault detection accuracies of 98% across varied loading circumstances [17]. Another study found that random forest classifiers were resilient to transient current response characteristics across operating circumstances [18]. As switching harmonics make fault detection difficult in inverter-fed IMs, Cyclic Modulation Spectral Analysis (CMSA) is adopted to separate fault-related frequencies from the inverter harmonics [19]. These spectrum methods improve clarity and SNR in complex systems, improving diagnostic reliability. In [17], authors proposed an enhanced BRB fault detection method for inverter-fed IM under various load conditions. However, with severely imbalanced datasets and varying load conditions, these approaches may lose accuracy, reducing potential practical applicability.

In addition, contrast estimation has become popular for BRB fault detection. This technique promotes fault visibility by analyzing current signal energy distribution fluctuations and is promising for real-time condition monitoring in low-noise situations [20]. Current advancements in the IM monitoring and diagnostics focus on attaining dependable failure identification without the need for supplementary thermal or mechanical sensors [21,22]. Building on the methodology established [23], research has progressed towards failure monitoring systems that amalgamate the physical model of the IM with a minimal sensor array, employing a Kalman filter to guarantee both precision and feasibility. Researchers proposed method [24] to identify broken bars, the data of stator voltages and currents are analyzed using an extended Kalman filter (EKF) for the simultaneous estimation of speed and rotor resistance. Kalman filter-based frameworks [25], encompassing adaptive and unscented variations, have enhanced the real-time detection of winding and rotor asymmetries. However, the accuracy may be diminished in the presence of higher noise levels and nonlinear operating situations.

Any diagnostic method relies on accuracy and sensitivity. A high accuracy helps distinguish fault-induced components from background noise, whereas sensitivity reflects a method’s capacity to detect early fault developments’ modest signal anomalies [26]. The accuracy and efficiency of these diagnostic methods depend diagnostic indicator sensitivity. A high accuracy makes fault-related signal components easier to spot. Instead, sensitivity indicators assess the system’s capacity to detect minor deviations from early-stage errors [27]. Signal processing, filtering, and contrast enhancement increase these qualities. These considerably enhance fault detection, especially in noisy or low-load environments [28]. To conclude, accuracy and sensitivity can be used to evaluate signal-based diagnostic approaches. This promotes more efficient and responsive maintenance tools. Notwithstanding these advancements, constraints persist regarding generalization across load and speed profiles, real-time monitoring of slip-dependent components, and the differentiation of fault-relevant harmonics from inverter-induced distortions.

It is worth noting that the application of the NSC for the diagnosis of the BRB has been explored in a limited number of studies, despite its evident diagnostic advantages over traditional MCSA. The NSC directly indicates rotor-induced asymmetries, although it is also acutely responsive to external disturbances, including supply imbalances, inherent machine asymmetries, and inverter-induced harmonics, which may hinder precise fault identification. This sensitivity presents the scarcity of NSC-based methodologies for BRB detection in the current literature.

This paper presents the development of the Spectrum-Tracking Hybrid Adaptive Extended Kalman Filter (ST-HAEKF) for the extraction of fault-related NSC components to identify BRB. The filter is applied to isolate the amplitudes of NSC at the fundamental frequency and the specific sideband frequencies linked to BRB faults, while simultaneously attenuating inverter-induced harmonics and other non-characteristic components unrelated to fault frequencies. This careful filtering guarantees the retention of only the most diagnostically relevant NSC components, enhancing the reliability and precision of fault diagnosis. Furthermore, the ST-HAEKF framework enables the approach to sustain high robustness across various load and speed conditions, while also improving both the accuracy and the sensitivity of the fault signature. The proposed method has been used for both healthy and faulty IMs, employing a baseline subtraction technique: the NSC profile derived from healthy operating conditions is deducted from the faulty condition, thereby facilitating a more distinct separation of the BRB-induced NSC components. This baseline-referenced comparison enhances the differentiation between healthy and faulty operations, yielding a more dependable Fault Detection Index that is robust against external disturbances and non-fault-related harmonics.

Our key research objectives were:

1. Development of the ST-HAEKF to extract fault-associated NSC components at fundamental and sideband frequencies, while efficiently suppressing inverter harmonics and other non-characteristic components.
2. Development of a Fault Detection Index (FDI) by deducting the healthy NSC baseline from defective motor circumstances, hence facilitating a more precise measurement of BRB fault signals across various load and speed conditions.
3. Thorough experimental validation of the NSC-based diagnostic methodology on both healthy and faulty motors, showcasing enhanced sensitivity, resilience to disturbances, suitability for real-time fault monitoring and their potential for incipient BRB fault detection.

This paper is organized as follows. Section 2 details the methodology, where an analytical model of the IM with a BRB fault and signal-processing techniques are introduced. Section 3 presents the experimental setup and signal acquisition. Section 4 discusses the results. Section 5 concludes the paper.

## 2. Methodology

This section presents an analytical model of the IM with a BRB fault and the signal-processing technique.

The captured signal should be treated by various signal-processing methods, such as filtering and feature extraction, to emphasize fault-related elements while minimizing harmonics. Quantitative metrics, including the accuracy and sensitivity values, were calculated to evaluate the clarity and strength of the retrieved features.

### 2.1. Analytical Model of the IM with BRB Fault

This subsection presents a mathematical description of the IM with the BRB fault, with definitions of fault severity, changes in rotor resistance and inductance, and the emergence of NSC sideband amplitude.

For a healthy IM, the voltage equations on the dq-axis are as follows [2]:

(1) $$\left\{\begin{array}{c} v_{ds}=R_{s}i_{ds}+\frac{d\lambda_{ds}}{dt}-\omega_{e}\lambda_{qs}\\ \begin{array}{c} v_{qs}=R_{s}i_{qs}+\frac{d\lambda_{qs}}{dt}+\omega_{e}\lambda_{ds}\\ 0=R_{r}i_{dr}+\frac{d\lambda_{dr}}{dt}-(\omega_{e}-\omega_{r})\lambda_{dr}\\ 0=R_{r}i_{qr}+\frac{d\lambda_{qr}}{dt}+(\omega_{e}-\omega_{r})\lambda_{qr} \end{array} \end{array}\right.$$

where *i_ds_*, *i_dr_* and *i_qs_*, *i_dr_* are the stator and rotor currents in the *dq*-frame, *R_s_* and *R_r_* are the stator and rotor resistances, and *ω_e_* and *ω_r_* are the electrical angular frequency and rotor speed. *λ_ds_*, *λ_dr_* and *λ_qs_*, *λ_qs_* are the stator and rotor flux linkages in the *dq*-frame, and they have:

(2) $$\left\{\begin{array}{c} \lambda_{ds}=L_{s}i_{ds}+L_{m}i_{dr}\\ \lambda_{qs}=L_{s}i_{qs}+L_{m}i_{qr}\\ \lambda_{dr}=L_{r}i_{dr}+L_{m}i_{ds}\\ \lambda_{qr}=L_{r}i_{qr}+L_{m}i_{qs} \end{array}\right.$$

where *L_S_*, *L_r_* are the stator and rotor inductances, and *L_m_* is the mutual inductance.

Electromagnetic torque:

(3) $$T_{e}=\frac{3}{2}\frac{P}{2}L_{m}(i_{dr}i_{qs}-i_{qr}i_{ds})$$

where *P* is the pole pair number.

Before describing the impact of BRB on motor parameters, the BRB fault severity (*δ*) is described as:

(4) $$\delta =A\cdot \frac{1}{N_{bars}}\sum_{k=0}^{N_{bars}-1}\chi_{k}\cdot \eta_{k}$$

where *A* is adjacency coefficient; *k* is bar index (from 0 to *N_bars_* − 1); *N_bars_* is the total number of rotor bars; *χ* is the weighting factor, which represents the spatial influence of bar *k*; *η* is the healthy state of bar *k* (1 = broken; 0 = healthy) and it can be represented as the binary array:

(5) $$\eta =[\eta_{0},\eta_{1},\ldots ,\eta_{N_{bars}-1}]$$

If two or more broken bars are adjacent, the effective severity is stronger than just their sum. The adjacency coefficient is defined as:

(6) $$A=1+\varepsilon \cdot \frac{N_{adj}}{N_{brb}}$$

where *N_adj_* is the number of adjacent broken bar pairs, *N_brb_* is the number of broken bars, and *ε* is weighting constant (identified through the experiment).

The bars of the rotor are designed to be geometrically circular. If bars are indexed *k* = 0, 1, 2, …, *N_bars_* − 1, then bar indexed “0” is next to bar *N_bars_* − 1. Thus, when adjacency is checked, it must be wrapped around the last bar and the first one.

(7) $$N_{adj}=\sum_{k=0}^{N_{bars}-1}\eta_{k}\cdot \eta_{(k+1)\mathrm{mod}N_{bars}}$$

The rotor is a circle with *N_bars_* uniformly spaced bars. The angular position of bar *k* is described as:

(8) $$\theta_{k}=\frac{2\pi k}{N_{bars}}$$

The spatial weighting factor in the proposed method is:

(9) $$\chi_{k}=1+\rho \cos(\frac{2\pi k}{N_{bars}})$$

where *ρ* controls how strongly position affects severity (0 < *ρ* < 1).

These equations above ensure that both the spatial location and adjacency of broken bars are included in severity measure.

A broken bar increases the effective rotor resistance *R_r_* because current must bypass the broken bar through adjacent bars and end-rings. Effective resistance becomes:

(10) $$R_{rf}=R_{r}+\Delta R_{r}=R_{r}(1+\delta )$$

where *R_r_* is the healthy rotor resistance, and Δ*R_r_* is the additional loss due to BRB.

(11) $$\Delta R_{r}=\delta \cdot R_{r}$$

The rotor leakage inductance depends on the distribution of currents in rotor bars and the resulting flux leakage paths. When a bar is broken, its current is forced to redistribute into adjacent bars and end-ring segments. This changes the local flux linkage, because some leakage flux paths become weaker, others stronger. The overall effective rotor leakage inductance increases.

(12) $$L_{rf}=L_{r}+\Delta L_{r}=L_{r}(1+\delta )$$

where *L_r_* is the healthy rotor inductance, and Δ*L_r_* is the additional loss due to BRB.

Mutual inductance represents the flux linkage shared between stator windings and rotor cage bars described in Equation (2). *L_m_* is governed primarily by the main air-gap flux, which depends on stator magnetizing current and iron core geometry. In a healthy machine, this flux is symmetrically distributed around the air-gap.

With the BRB, the air-gap magnetomotive force (MMF) distribution is no longer sinusoidal, equivalent to having a space-harmonic perturbation:

(13) $$F(t)=F_{1}\cos(2\pi f_{s}t-\phi )+F_{brb}\cos(2\pi sf_{s}t+\phi )$$

The first term is positive-sequence MMF (healthy fundamental). The second term is negative-sequence MMF proportional to fault severity *δ*. This extra component modulates the effective stator–rotor coupling and mutual inductance becomes:

(14) $$L_{mf}(t)=L_{m}+\Delta L_{m}\cos(2\pi \cdot 2sf_{s}\cdot t+\phi )$$

where the modulation frequency is 2*sf* (twice slip frequency), and the amplitude Δ*L_m_* depends on severity *δ*.

(15) $$\Delta L_{m}=L_{m}\cdot \delta$$

Substituting mutual inductance in Equations (14) and (15) for the BRB fault becomes:

(16) $$L_{mf}(t)=L_{m}(1+\delta \cos(4\pi sf_{s}t+\phi ))$$

As mentioned above, the missing bar introduces a space-harmonic MMF distortion which leads to negative-sequence components and space harmonics in the stator current. Specifically, the BRB fault creates sidebands around the supply (fundamental) frequency:

(17) $$f_{BRB}=(1\pm 2s)f_{s}$$

Thus, NSC due to the BRB fault is described as:

(18) $$i_{2-brb}=I_{2-f_{s}}\sin(2\pi f_{s}t+\phi_{2})+I_{2-ls}\sin((1-2s)2\pi f_{s}t+\phi_{1})+I_{2-us}sin((1+2s)2\pi f_{s}t+\phi_{3})$$

where *I*_2−fs_, *I*_2−ls_, and *I*_2−us_ are amplitudes of NSC at fundamental frequency, lower-sideband frequency, and upper-sideband frequency, respectively; *f*_s_—fundamental frequency; *ϕ*_1_, *ϕ*_2_, *ϕ*_3_ are corresponding phases.

The sideband amplitude of NSC can now be expressed as:

(19) $$I_{2-sb}\approx \frac{R_{r}\delta }{\left(1+\delta )({R_{r}}^{2}+4\pi^{2}f_{s}^{3}s^{2}L_{r}^{2}\right)}I_{s}$$

**Figure 1:**
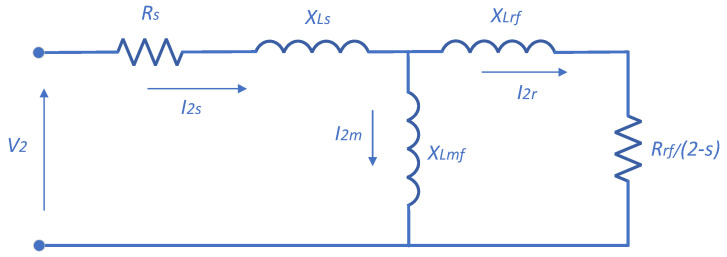
Negative-sequence equivalent circuit of the IM with the BRB fault.

The frequency of the NSC in the rotor of the IM is expressed as (2 − *s*)*f_s_*. The NSC generates a field that rotates in opposition to the primary field. The negative-sequence field generates oscillations in the torque and power of the induction motor at double the supply frequency. The negative-sequence equivalent circuit is depicted in Figure 1.

### 2.2. Signal Proccesing

#### 2.2.1. Spectrum Tracking–Hybrid Adaptive Extended Kalman Filter

The predominant methods of MCSA are founded on the principle that faults in electrical machines generate specific frequency components in the current spectrum. These harmonics act as diagnostic signatures, facilitating early fault identification. Various machine anisotropies, including those arising from manufacturing or assembly imbalances, can produce distinct frequency components in the stator current. The frequency components associated with BRBs can be articulated as Equation (17). This harmonic generates two distinct sidebands. The LSB results from the unbalanced magnetic forces generated by the asymmetrical distribution of rotor bars, whereas the USB is a consequence of speed oscillations that arise from periodic variations in torque [29].

The characteristics of supply-side harmonics in electrical drive systems are mostly determined by the power source. In grid-connected systems, the harmonic content is often restricted to the odd multiples of the basic supply frequency. When the machine is powered by an inverter, the spectrum significantly broadens, incorporating a diverse array of high-frequency components. In inverter-fed IMs, the switching activities of inverter operation led to supplementary power losses in both the switching devices and the machine magnetic core, as well as torque ripple, acoustic noise, and electromagnetic interference. Thus, whereas inverters facilitate improved dynamic performance and precise regulation of electrical machines, they also complicate system behavior by introducing a heterogeneous and extensive dispersion of harmonics within the current spectrum. This has prompted significant research [30,31] into sophisticated control schemes and optimal switching approaches designed to reduce harmonic distortion and enhance overall drive performance, although frequently at the expense of increased system complexity.

To precisely identify a BRB fault, it is crucial to observe both the fault-specific frequency components and the related amplitudes of NSC linked to rotor asymmetry. The suggested ST-HAEKF method isolates the BRB fault signature from other harmonic components and extraneous frequencies by accurately monitoring the slip-dependent sideband frequencies and their NSC amplitude fluctuations. This targeted monitoring facilitates precise detection of the BRB fault frequency and its NSC magnitude, reducing interference from supply or inverter-generated harmonics. The ST-HAEKF guarantees elevated sensitivity and resilience in fault detection, with a comprehensive implementation outlined in this subsection.

The proposed ST-HAEKF primarily reproduces the negative-sequence phasors at the three specific fault-related frequencies. Inverter-induced harmonics manifest at frequencies excluded from the state model, so they are regarded as unmodeled dynamics and incorporated into the process/measurement noise covariance. The filter intrinsically eliminates inverter-related imbalances while only monitoring the phasors linked to the faults.

The NSC phasors at the fundamental frequency *f_s_*, the sidebands (1 − 2*s*)*f*_s_ and (1 + 2*s*)*f*_s_ are estimated using cosine/sine quadrature shown in Equation (51). An additional state slip *s* allows the filter slip to be adaptive in response to variations in speed and load. This directly parameterizes the fault-salient spectrum while maintains compactness.

(20) $$x_{k}={\left[\begin{array}{ccccccc} i_{2-1k} & i_{2-2k} & i_{2-3k} & i_{2-4k} & i_{2-5k} & i_{2-6k} & s_{k} \end{array}\right]}^{T}$$

where

(21) $$\left\{\begin{array}{c} i_{2-1k}=I_{2-1k}\cos\theta_{F}\\ i_{2-2k}=I_{2-1k}\sin\theta_{F}\\ i_{2-3k}=I_{2-2k}\cos\theta_{LSB}\\ i_{2-4k}=I_{2-2k}\sin\theta_{LSB}\\ i_{2-5k}=I_{2-3k}\cos\theta_{USB}\\ i_{2-6k}=I_{2-3k}\sin\theta_{USB} \end{array}\right.$$

The measured negative-sequence component (*z_k_*) is the sum of the three tracked cosine projections; ν*_k_* ∼ *N*(0, *R_k_*) represents inverter-induced noise, which the EKF models statistically. Using a linear measurement keeps the update simple and numerically robust. Then, it has:

(22) $$z_{k}=h(x_{k})+v_{k}$$

where

(23) $$h(x_{k})=H_{k}\cdot x_{k}$$

The Jacobian measurement is:

(24) $$H_{k}=\frac{\partial h(x_{k})}{\partial x_{k}}=[\begin{array}{ccccccc} 1 & 0 & 1 & 0 & 1 & 0 & 0 \end{array}]$$

Here, “1” corresponds to cosine quadratures of NSC at the given frequencies, which directly project onto the *d*-axis. “0” represents the sine quadratures which do not contribute to the *d*-axis measurement, but they are necessary in the state model for proper phasor rotation. The seventh and last “0” is for slip, and not directly measurable. It only affects dynamics, not the instantaneous *d*-axis measurement.

This compact form shows that the ST-HAEKF measurement update corrects the phasor cosine terms directly, while the sine terms and slip are corrected indirectly through the state dynamics.

The fundamental negative-sequence phasor rotates at the supply angular frequency *ω_s_* = *2πf_s_*, which is known and fixed (set by the inverter). The lower and upper sidebands rotate at the frequencies (1 − 2*s*)*f_s_* and (1 + 2*s*)*f_s_*. Their position depends on the slip. Estimating slip ensures both sidebands are tracked consistently even under varying load.

(25) $$\left\{\begin{array}{c} i_{2-1k}=-\omega_{s}i_{2-2k}\\ i_{2-2k}=\omega_{s}i_{2-1k}\\ i_{2-3k}=-(1-2s_{k})\omega_{s}i_{2-4k}\\ i_{2-4k}=(1-2s_{k})\omega_{s}i_{2-3k}\\ i_{2-5k}=-(1+2s_{k})\omega_{s}i_{2-6k}\\ i_{2-6k}=(1+2s_{k})\omega_{s}i_{2-5k} \end{array}\right.$$

Slip varies slowly, driven by changes in load torque, rotor condition, and speed. The process noise *w_k_* allows the filter to adjust the slip flexibly when operating conditions change. This is essential for fault detection, since BRB faults manifest precisely through changes in slip-modulated sidebands.

(26) $$s_{k}=w_{k}\;\;\;\;\;\;\;\;\;\;\;\;w_{k}∼N((0,\;q_{s}T_{s})$$

Slip update:

(27) $$s_{k+1}=s_{k}+w_{k,s}\;\;\;\;\;\;\;\;\;\;\;\;w_{k,s}∼N((0,\;q_{s}T_{s})$$

The Jacobian state (*F_k_*) captures both phasor rotation and sensitivity to slip.

(28) $$F_{k}=\frac{\partial f}{\partial x}{|}_{{\hat{x}}_{k}}$$

(29) $$\left\{\begin{array}{c} \frac{\partial i_{2-3k}}{\partial s}=2\omega_{s}i_{2-4k}\\ \frac{\partial i_{2-4k}}{\partial s}=-2\omega_{s}i_{2-3k}\\ \frac{\partial i_{2-5k}}{\partial s}=-2\omega_{s}i_{2-6k}\\ \frac{\partial i_{2-6k}}{\partial s}=2\omega_{s}i_{2-5k} \end{array}\right.$$

This means that any error in slip estimation directly affects the predicted sideband states. By correcting slip online, the filter keeps the sideband phasors aligned with the real machine behavior. Then, it has:

(30) $$F_{k}=\left[\begin{array}{ccccccc} 0 & -\omega_{s} & 0 & 0 & 0 & 0 & 0\\ \omega_{s} & 0 & 0 & 0 & 0 & 0 & 0\\ 0 & 0 & 0 & -(1-2s_{k})\omega_{s} & 0 & 0 & 2\omega_{s}i_{2-4k}\\ 0 & 0 & (1-2s_{k})\omega_{s} & 0 & 0 & 0 & -2\omega_{s}i_{2-3k}\\ 0 & 0 & 0 & 0 & 0 & -(1+2s)\omega_{s} & -2\omega_{s}i_{2-6k}\\ 0 & 0 & 0 & 0 & (1+2s_{k})\omega_{s} & 0 & 2\omega_{s}i_{2-5k}\\ 0 & 0 & 0 & 0 & 0 & 0 & 0 \end{array}\right]$$

In this matrix, rows 1–2 (fundamental), rotation at *ω_s_*. No dependence on slip, since slip does not affect the fundamental negative sequence. Rows 3–4 (lower sideband): Rotation at (1 − 2s)ω_s_. The last column entries are the derivatives of the slip. Rows 5–6 (upper sideband): Rotation at (1 + 2s)ω_s_. Again, slip sensitivity shows up in the last column. Row 7 (slip): Random walk-zero dynamics, so the last row is comprised of zeros.

It is essential to first provide the specifics of the standard extended Kalman filter (SEKF), adaptive fading extended Kalman filter (AFEKF), and innovation-based adaptive extended Kalman (IAEKF) to comprehend the functionality of the proposed ST-HAEKF.

An SEKF can be expressed by the following equations [32].

Initialization

(31) $${\hat{x}}_{0}=E\;[x_{0}],\;\;\;\;\;\;\;\;\;\;P_{0}=\;E\;[(x_{0}-{\hat{x}}_{0}){\left(x_{0}-{\hat{x}}_{0}\right)}^{T}]$$

Time Update:

(32) $${\hat{x}}_{k}^{-}=f({\hat{x}}_{k-1}),\;\;\;\;\;\;\;\;\;\;P_{k}^{-}=(I_{7x7}+F_{k-1}T_{s})P_{k-1}{\left(I_{7x7}+F_{k-1}T_{s}\right)}^{T}+Q_{k-1}$$

Measurement Update:

(33) $$K_{k}=P_{k}^{-}H_{k}^{T}{\left(H_{k}P_{k}^{-}H_{k}^{T}+R_{k}\right)}^{-1}$$

(34) $${\hat{x}}_{k}={\hat{x}}_{k}^{-}+K_{k}(z_{k}-H_{k}{\hat{x}}_{k}^{-})$$

(35) $$P_{k}=(I_{7x7}-K_{k}H_{k}){P_{k}}^{-}{\left(I_{7x7}-K_{k}H_{k}\right)}^{T}+K_{k}R_{k}K_{k}^{T}$$

In contrast to SEKF, AFEKF addresses the negative impact of the incomplete dynamic equation by applying a forgetting factor to the error covariance matrix in (32). The influence of older data in the filtering process is diminished [33].

Covariance prediction with forgetting factor:

(36) $${P_{k}}^{-}=\lambda_{k}[(I_{7x7}-F_{k}T_{s})P_{k-1}{\left(I_{7x7}-F_{k}T_{s}\right)}^{T}+Q_{k-1}]$$

The optimal forgetting factor can be derived by

(37) $$\lambda_{k}=\max\{1,\;\frac{tr(N_{k})}{tr(M_{k})}\}$$

Under the following assumptions:

(1) *Q_k_*, *R_k_*, and *P*_0_ are positive definite.

(2) The measurement matrix *H_k_* has full rank.

*M_k_* and *N_k_* matrices are

(38) $$M_{k}=H_{k}(I_{7x7}+F_{k-1}T_{s})P_{k-1}{\left(I_{7x7}+F_{k-1}T_{s}\right)}^{T}H_{k}^{T}$$

(39) $$N_{k}=C_{0,k}-R_{k}-H_{k}Q_{k-1}H_{k}^{T}$$

where

(40) $$C_{0,k}=\left\{\begin{array}{c} v_{0}v_{0}^{T},\;\;\;\;\;\;\;\;\;\;\;\;\;\;k=0\\ \frac{2}{1+\lambda_{k}}v_{k}v_{k}^{T},\;\;\;\;\;\;k\geq 1 \end{array}\right.$$

(41) $$v_{k}=z_{k}-H_{k}{\hat{x}}_{k}^{-}$$

AFEKF inflates uncertainty during abrupt changes (*λ_k_*  >  1), so the estimator trusts new data more, improving convergence at fault onset, load steps, or speed reversals.

IAEKF learns *Q* from data via the innovations, removing manual tuning and tracking operating-point changes (incl. inverter-harmonic background). SEKF and AFEKF use the predefined *Q* and *R* matrices, which are assumed to be diagonal and are usually tuned empirically. Contrary to SEKF and AFEKF, the IAEKF provides full-covariance estimation and does not require a tuning effort [34]. The primary structure of the IAEKF mirrors that of the SEKF, with the exception of the fixed *Q_k_* assumption. The simultaneous estimation of both noise covariance matrices may result in divergence issues. Empirical evidence indicates that *Q* is more effective for the specified problem. The covariance estimation of the innovation residual can be derived by averaging the previous residual sequences over an *N*-length moving window as follows:

Windowed innovation covariance

(42) $${\hat{C}}_{v,k}=\frac{1}{N}\sum_{j=k-N+1}^{k}v_{j}v_{j}^{T},\;\;\;\;\;\;\;\;\;\;\;\;v_{j}=z_{j}-H_{j}{\hat{x}}_{j}^{-}$$

Process-noise update

(43) $${\hat{Q}}_{k}=\frac{1}{N}\sum_{j=k-N+1}^{k}\Delta x_{j}\Delta x_{j}^{T}+P_{k}-(I_{7x7}+F_{k-1}T_{s})P_{k-1}{\left(I_{7x7}+F_{k-1}T_{s}\right)}^{T}$$

(44) $$\Delta x_{j}={\hat{x}}_{j}-{\hat{x}}_{j}^{-}$$

(45) $${\hat{C}}_{v,k}={\hat{C}}_{v,k-1}\frac{1}{N}(v_{k}v_{k}^{T}-v_{k-N}v_{k-N}^{T})$$

The selections of the forgetting factor and the duration of the sliding window are important to the operation of the ST-HAEKF, as these parameters govern the filter’s sensitivity to dynamic variations and its adaptability to inverter and measurement noise. The forgetting factor amplifies the estimated state covariance presented in Equation (36), thereby causing the filter to assign greater significance to recent data. This enhances swift tracking during unexpected shifts, such as load or speed changes and fault, while diminishing the impact of outdated data. The theoretical justification for the optimal factor, based on the specified assumptions, is presented in Equation (37). The forgetting factor enhances adaptability but may increase the noise of measurements and result in increased steady-state estimation variance, whereas small forgetting factor values produce smoother estimation but reacts more slowly to actual changes. For inverter-fed IMs functioning under standard industrial variability, the forgetting factor value between 1.00 and 1.20 is often effective.

The window length *N* in Equation (42) indicates the number of earlier innovation samples utilized to calculate the average innovation covariance and update the process noise *Q*. A larger window enhances the stability of the covariance estimate, reducing variability under consistent conditions; however, it requires more time to adjust to actual changes. A reduced timeframe responds more rapidly but yields a more erratic estimate. So, *N* should be selected to ensure the window encompasses a specified number of electrical cycles of the fundamental frequency. This establishes a direct correlation between *N*, the sampling frequency, and the supply frequency.

The most effective forgetting factor and *N* have minor dependence on the motor parameters, such as number of bars and winding configuration, which influence the spectral distribution of sideband components. However, the proposed ST-HAEKF explicitly estimates the slip and the NSC components according to innovation-based adaptation filter, which intrinsically reduces sensitivity to geometric differences, enabling the filter to sustain strong performance across various motor configurations without necessitating complete retuning.

When several adjacent rotor bars are broken, the subsequent asymmetry becomes widely spread out, resulting in the increase in total NSC amplitude but with diminished specific sidebands. The proposed ST-HAEKF remains effective, as it executes dynamic state estimation. To augment adaptability, the forgetting factor may be somewhat elevated and the sliding window duration lowered to enhance responsiveness to modest yet persistent BRB fault-induced asymmetry. Thus, the proposed ST-HAEKF exhibits strong fault tracking and dependable detection capabilities in both single- and multiple-bar fault situations without any fundamental alterations to the algorithm.

#### 2.2.2. Framework

**Figure 2:**
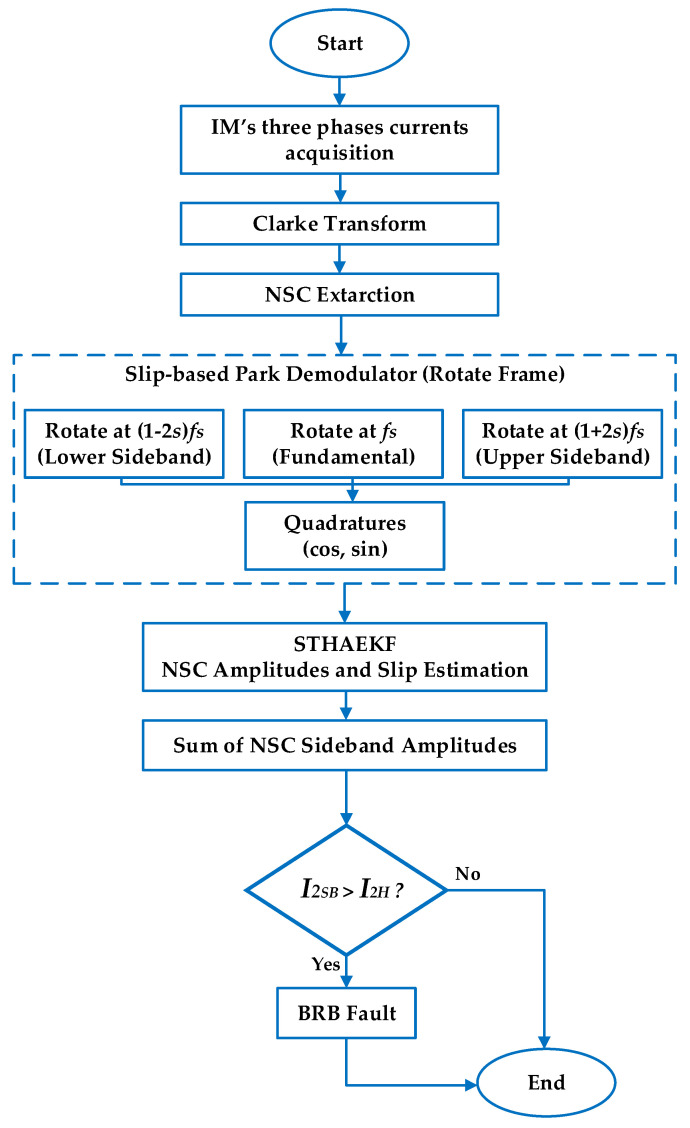
Flowchart of proposed method.

This subsection outlines the proposed diagnostic framework illustrated in Figure 2. The process starts with the simultaneous capture of three-phase stator current signals, which are subsequently converted into the αβ reference frame via the Clarke transform to eliminate zero-sequence components and facilitate data processing. The NSC is subsequently extracted to emphasize rotor asymmetry induced by a BRB fault. The ST-HAEKF algorithm subsequently follows the NSC components at fundamental and slip-dependent sideband frequencies, while predicting motor slip and filtering inverter-induced harmonics via covariance adaptation. The collected NSC sidebands amplitudes constitute a Fault Detection Index (FDI), which is evaluated against a threshold established from the healthy baseline to ascertain the motor’s state.

The three-phase stator currents of the IM are synchronously acquired and transformed to the orthogonal *αβ* frame using the Clarke transform, which eliminates the zero-sequence component and prepares the data for further processing.

(46) $$\left[\begin{array}{c} i_{\alpha }\\ i_{\beta } \end{array}\right]=\left[\begin{array}{ccc} \frac{2}{3} & -\frac{1}{3} & -\frac{1}{3}\\ 0 & \frac{1}{\sqrt{3}} & -\frac{1}{\sqrt{3}} \end{array}\right]\left[\begin{array}{c} i_{a}\\ i_{b}\\ i_{c} \end{array}\right]$$

The space vector of the current:

(47) $$i_{s}(t)=i_{\alpha }(t)+ji_{\beta }(t)$$

where *i_a_*, *i_b_*, *i_c_* are three-phase currents of IM. In three-wire machines, *i*_0_ = 0.

The NSC is derived from the *αβ* currents through symmetrical-components relations, highlighting rotor asymmetry and minimizing the influence of the dominant balanced (positive-sequence) fundamental.

Within the complex *αβ*-frame, the current is broken down into pieces that rotate in the opposite direction:

(48) $$i_{s}(t)=I_{1}e^{j(\omega_{s}t+\phi_{1})}+I_{2-1}e^{-j(\omega_{s}t+\phi_{1})}+I_{2-2}e^{-j((1-2s)\omega_{s}t+\phi_{2})}+I_{2-3}e^{-j((1+2s)\omega_{s}t+\phi_{3})}+I_{2}(t)$$

where *I*_1_ is the positive-sequence current; *I*_2−1_, *I*_2−2_, *I*_2−3_ are the NSC amplitudes at the fundamental, LSB, USB frequencies, respectively; *s* is the slip; *I*_2_(*t*) is the sum of NSCs due to inverter switching frequencies and their associated sidebands, PWM ripple, quantization and aliasing effects, grid distortion, slot and winding harmonics, eccentricity components, sensor noise, offsets and drifts.

Therefore, the NSC is the component rotating at −*ω_s_*. This method utilizes approach by extracting the NSC channel, effectively projecting onto the clockwise subspace. This process suppresses the predominant positive-sequence content, allowing the asymmetry-related component to be processed in the next step. This step increases sensitivity to BRB, as BRB-induced currents primarily manifest in the negative sequence and its slip-dependent sidebands.

The NSC channel undergoes demodulation within three slip-adaptive Park frames, with angular speeds configured to align with the fundamental and BRB sidebands. This rotation transforms each fault-related tone into a quasi-DC pair of cosine and sine quadratures, effectively encoding its amplitude and phase in a compact manner.

Park represents a rotation of the *αβ* frame by an angle *θ*(*t*) = ∫*ω*(*t*)*dt*.

(49) $$\left[\begin{array}{c} i_{dk}\\ i_{qk} \end{array}\right]=\left[\begin{array}{cc} \cos\theta_{k} & \sin\theta_{k}\\ -\sin\theta_{k} & \cos\theta_{k} \end{array}\right]\left[\begin{array}{c} i_{\alpha }\\ i_{\beta } \end{array}\right]$$

Demodulation angles of fundamental *θ_F_*(*t*), lower sideband *θ_LSB_*(*t*) and upper sideband *θ_USB_*(*t*) frequency to track slip are followings:

(50) $$\left\{\begin{array}{c} \theta_{F}(t)=\omega_{s}t\\ \theta_{LSB}(t)=(1-2s)\omega_{s}t\\ \theta_{USB}(t)=(1+2s)\omega_{s}t \end{array}\right.$$

*i_s_* has a negative-sequence tone at *ω_k_* equivalent to the selected value of *θ_k_*.

(51) $$\left\{\begin{array}{c} i_{dk}=I_{k}\cos\theta_{k}\\ i_{qk}=I_{k}\sin\theta_{k} \end{array}\right.$$

The NSC components due to BRB revolve in a clockwise direction at sidebands. Subsequently, rotate the frame by the aforementioned slip-adaptive angles to render those components direct current; the demodulated quadratures provide the NSC amplitudes and phases directly.

The quadratures, along with slip, constitute the state of the proposed ST-HAEKF, which simultaneously estimates the NSC amplitudes at the lower sideband, fundamental, and upper sideband, in addition to the slip. Innovation-based adaptation effectively mitigates non-characteristic inverter harmonics and ensures tracking during speed and load transients. The proposed ST-HAEKF integrates the benefits of both AFEKF and IAEKF. The covariance scaling mechanism in AFEKF is utilized to enhance estimation accuracy. Performance during dynamic changes in IM states and the full-covariance estimation mechanism in IAEKF for online tuning of *Q_k_*.

Unlike conventional FFT-based diagnosis, where inverter-induced harmonics appear alongside fault signatures, the proposed ST-HAEKF inherently isolates fault-related NSC. The state model explicitly tracks only the phasors at (1 − 2*s*)*f_s_*, *f_s_*, and (1 + 2*s*)*f_s_*. Any negative-sequence components arising from inverter harmonics at non-characteristic frequencies are not represented in the model and therefore absorbed into the process and measurement noise. This enables the filter to suppress inverter-related imbalance and provide amplitudes that are exclusively fault-sensitive.

A decision feature is established as the aggregate of the estimated NSC sideband amplitudes and is compared against a threshold determined from a healthy baseline, such as a statistically selected quantile. If the sum of the amplitudes of NSC sidebands exceeds the threshold, a BRB condition is identified; otherwise, the machine is deemed healthy.

## 3. Experimental Setup and Data Acquisition

This section presents a comprehensive description of the experimental configuration utilized for BRB fault detection and illustrates the techniques implemented for data collection. The laboratory testbench was built to imitate real conditions for operation of a three-phase IM in both healthy and faulty states.

### 3.1. Experimental Setup

**Figure 3:**
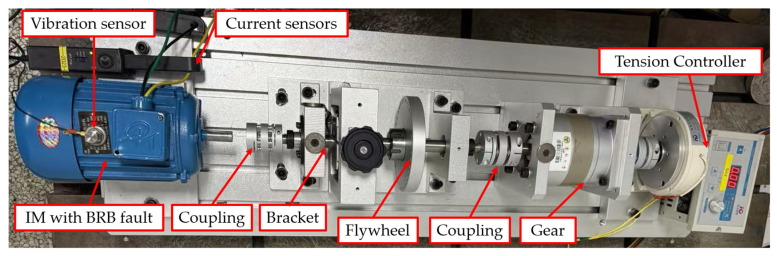
Complete laboratory-based experimental setup.

The setup shown in Figure 3 involved two identical squirrel-cage induction motors configured as follows:

Motor 1 (Healthy): Operated in standard factory condition with no structural or electrical faults.

**Figure 4:**
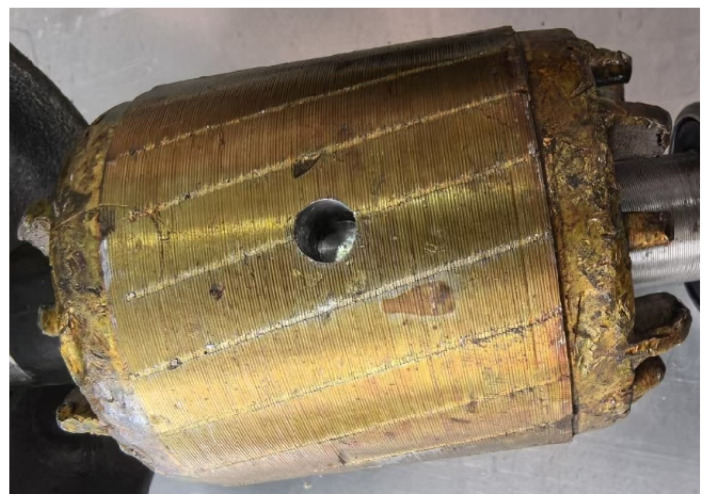
The squirrel-cage rotor with one broken bar for experimental setup.

Motor 2 (Faulty): Intentionally modified to include a single BRB to emulate the fault scenario. The rotor with one BRB for experiment is shown in Figure 4.

Each motor was independently connected to a variable mechanical load, ensuring that both were subjected to identical operational and environmental conditions throughout the testing process. The motor’s parameter is provided in Table 1.

### 3.2. Data Acquisition

To setup a range of real-world industrial scenarios, both motors were tested across: Supply frequencies: 10 Hz (low speed condition) and 50 Hz (high speed condition); Load levels: 0%, 50%, and 100% of the rated torque.

Three-phase currents are collected using current sensors. Then, NSC is extracted using proposed methodology. The negative sequence reflects the asymmetry in the rotor circuit caused by broken bars, even under a balanced three-phase supply.

All signals were sampled at a high rate (10 kHz or higher) and recorded for a consistent time duration (10 s) per test point.

**Figure 5:**
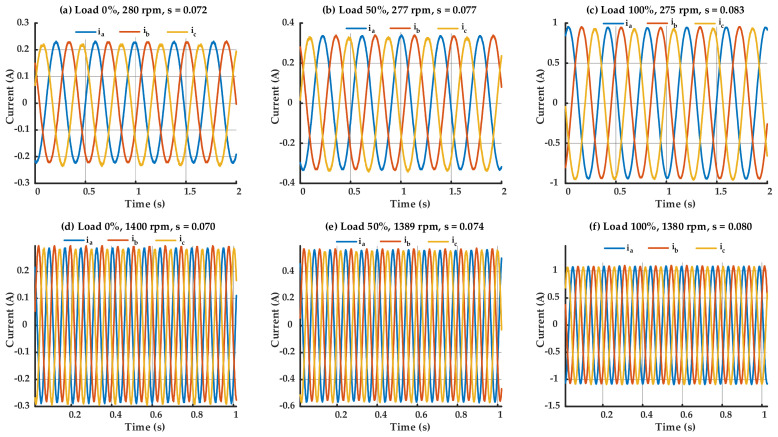
Three-phase currents waveform of the healthy IM.

**Figure 6:**
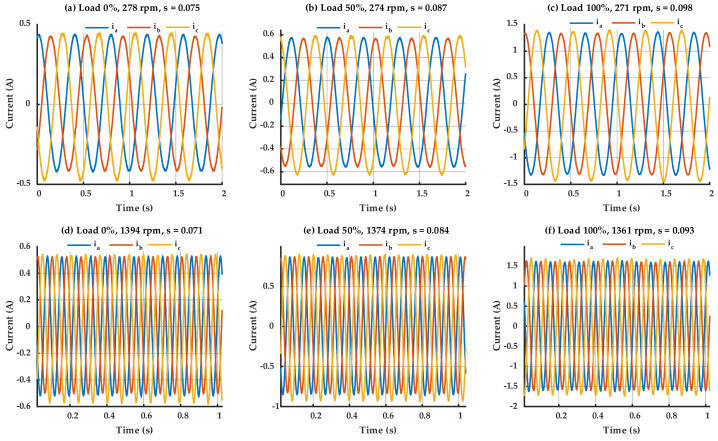
Three-phase currents waveform of the IM with one BRB.

Although operating under healthy conditions, the three-phase current waveforms depicted in Figure 5 demonstrate a minor yet visible imbalance among the phases. This small imbalance is characteristic of real-world IMs and can be related to variables such as magnetic irregularities or harmonics created by inverters. Conversely, the waveforms depicted in Figure 6, associated with the IM having a BRB, demonstrate markedly higher imbalance and distortion, especially under high load conditions. It can be seen from Figure 5 and Figure 6 that the speed and the slip of the IM are being changed according to the load levels, even under same power supply frequency. Nevertheless, the similarity in forms between healthy and faulty signals—particularly at low loads—makes the detection of rotor faults based exclusively on time-domain waveforms impractical. The differences are complicated, and no clear indicator of the BRB can be consistently detected in the time domain alone. Consequently, sophisticated diagnostic technique such as frequency-domain analysis is crucial for the precise identification and isolation of the BRB fault.

The studies were conducted under three load and two speed (power supply frequency) conditions. Each test was conducted three trials for both the healthy IM and the IM having the BRB fault, concluding in a total of 36 tests.

## 4. Results and Discussions

This section describes and evaluates the experimental outcomes derived from the proposed methodology. Priority is directed towards assessing the system’s performance under varying operational conditions, specifically concentrating on the identification and diagnosis of BRB using NSC analysis.

### 4.1. Negative Sequence Current Signal Analaysis

**Figure 7:**
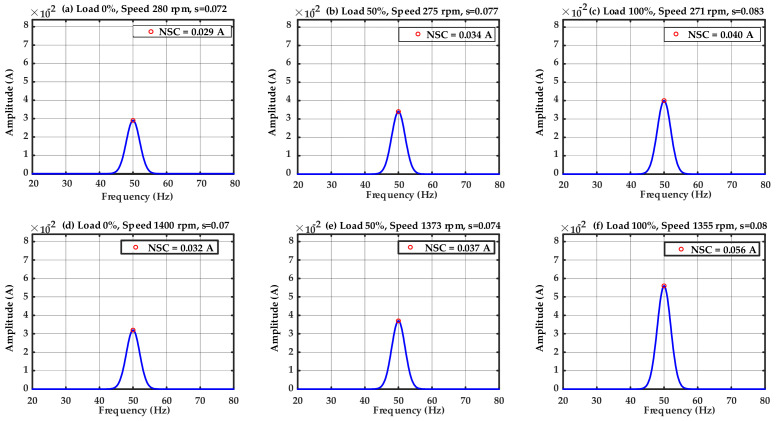
Spectrum of NSC for the healthy IM.

Inherent asymmetries in the motor and drive system make the achievement of accurately balanced and ideal operating conditions unfeasible. Consequently, a minor NSC invariably exists, even when the IM is healthy. Alongside these inevitable asymmetries, the suboptimal attributes of the voltage source inverter (VSI)—such as PWM and limited switching frequencies—induce harmonic distortion that exacerbates the residual NSC. The harmonics, along with structural defects, interact with the motor’s symmetrical components to generate a slight yet quantifiable NSC. In contrast to voltage imbalance, which is externally forced, inverter-induced harmonics and intrinsic asymmetries are inherent to the system and so persist even in balanced and optimal operating conditions. Figure 7 demonstrates that the resultant NSC amplitude is minimal (less than 0.1 A), after proposed ST-HAEKF method is applied, indicating the baseline effects of inherent asymmetries, rather than suggesting any motor malfunction. The subplots a-c show the NSC amplitude at low speed (10 Hz of supply frequency) whereas the subplots d-e show the amplitude of NSC at high speed condition (50 Hz).

**Figure 8:**
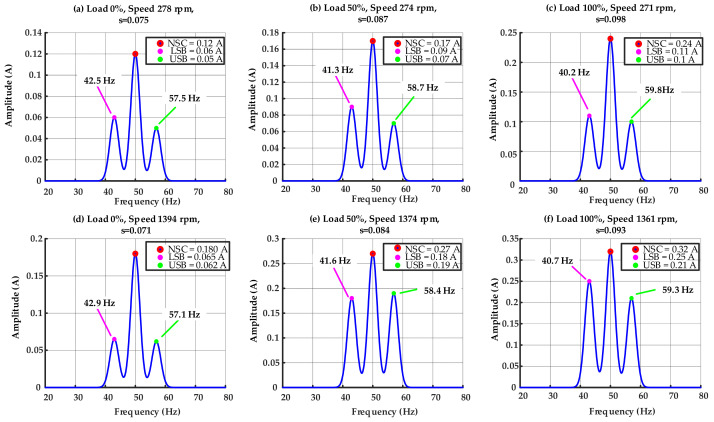
Spectrum of the NSC for the IM with one BRB.

Figure 8 shows the NSC spectra of the IM exhibiting one BRB fault across different speed and load conditions. The subplots a–c represent operating at a 10 Hz supply frequency (low-speed condition), whereas subplots d–f represent the 50 Hz supply frequency (high-speed condition). Tests were conducted at 0%, 50%, and 100% load for each supply frequency to assess the impact of mechanical loading on fault signs. Under no load shown in subplots a and d, the rotor speed approaches synchronous velocity, yielding minimal slip and feeble sideband components. As the load escalates (b, e), the slip increases, resulting in more pronounced fault-related sidebands at (1 ± 2*s*). Under full load shown in subplots c and f, the slip markedly increases, and the sidebands, around 40 Hz and 59 Hz, become predominant, indicating that BRB faults are more pronounced under elevated torque requirements. Moreover, a comparison of the two supply frequencies indicates that at 50 Hz, the fault signals are more evident than at 10 Hz, attributable to the synergistic effects of elevated synchronous speed and increased load torque. Figure 8 illustrates the combined effect of speed condition and mechanical load on slip variation and sideband proliferation in the context of BRB fault.

In the healthy motor, it is noteworthy that such sidebands were absent, and only a minimal NSC (less than 0.1 A) was detected, owing to intrinsic asymmetries. The presence of unique and elevated LSB of the NSC and USB of the NSC components in the faulty case clearly indicates the BRB fault. Moreover, the amplitude variation among the subplots indicates that an increase in load and speed amplifies the sideband strength, whereas under lighter load conditions, the sidebands remain weaker yet still discernible.

It is worth noting, Figure 8 illustrates that the fundamental component of the NSC escalates when a rotor bar is broken. The increase arises from rotor asymmetry, which disrupts current distribution and manifests in the stator as unbalanced impedance, hence creating distortion at the supply frequency. But the sideband components of the NSC, directly associated with the slip-frequency modulation caused by the BRB, are regarded as the primary and sole fault indications in this analysis.

Furthermore, Figure 8 illustrates that the proposed ST-HAEKF method guarantees precise tracking and estimate of slip and the NSC at both the fundamental frequency and the fault-related sideband frequencies. This real-time functionality facilitates dependable observation of slip progression under speed and load variations.

Table 2 summarizes the NSC amplitudes of both healthy and faulty IMs obtained by experimental results throughout varying operational conditions. A minor yet non-negligible NSC is detected in the healthy motor, predominantly at the fundamental frequency, resulting from inherent asymmetries in the machine, rendering the attainment of an ideally zero NSC unfeasible. Conversely, faulty states yield markedly greater NSC amplitudes, as the overall faulty NSC include contributions from both the characteristic sideband frequencies generated by the BRB fault. The findings indicate that NSC amplitude escalates with both load and speed, attaining its peak under high-speed and full-load conditions. To enhance fault sensitivity, the intact baseline NSC may be deducted from the faulty NSC measurement, thereby yielding a more distinct fault-related signal. This subtraction mitigates the impact of intrinsic system flaws, consequently elevating detection sensitivity, and improving overall fault diagnosis precision. The findings validate that the suggested filtering and estimating ST-HAEKF method proficiently monitors and differentiates between healthy and faulty states, while underscoring the reliability of NSC as a fault indicator.

The healthy baseline NSC serves as the threshold for fault identification. Given that tiny but nonzero NSC values consistently exist at the fundamental frequency due to intrinsic asymmetries, contrasting the observed NSC with this baseline enables a definitive differentiation between healthy and faulty states. When the NSC amplitude in operation beyond the acceptable threshold, the higher component can be related to fault-induced asymmetries, rendering the indication unequivocal and straightforward to understand. This method improves the accuracy, increases detection sensitivity, and minimizes false alarms by considering typical fluctuations in the motor. Establishing the healthy baseline as the threshold streamlines the diagnostic process and offers a reliable and effective NSC-based fault detection approach. Moreover, our experimental findings indicate that the ST-HAEKF method effectively confirms fault detection, even in demanding low-speed and no-load settings, underscoring its efficacy across many operational scenarios.

According to our experimental results, a threshold of 0.03 A has been established. When the sum of the sideband amplitudes of the NSC is equal to or greater than 0.03 A, this indicates the presence of the BRB fault. In this study, the detection threshold is set by utilizing the statistical properties of the healthy baseline condition to guarantee consistent and reliable performance assessment. The threshold setting procedure is applicable during the initial tuning of the proposed method to the motor. While prolonged motor aging may progressively alter the statistics of a healthy state, regular recalibration of the healthy baseline during maintenance intervals can mitigate the effects of aging, enabling the initial threshold-setting framework to remain applicable without necessitating further algorithmic adjustments.

It is worth noting that in some studies, a partial broken bar fault in the IM is studied, instead of fully developed BRB. The incipient faults, such as partial BRB, generate modest asymmetries that are challenging to be precisely detected. In order to assess the sensitivity and effectiveness of the proposed diagnostic methodology in early-stage fault scenarios, we perform simulation-based investigations under an incipient BRB fault with 25% damage in one rotor bar.

In Table 3, the NSC values for healthy motor are based on the experimental test, and the NSC values for the faulty motor are the simulation outcomes. An incipient fault in BRB, characterized by around 25% damage to one rotor bar, is modeled by setting the BRB fault severity *δ* = 0.25. Table 3 demonstrates that, under various operational conditions, the NSC amplitude in the motor with partial BRB consistently exceeds that of the healthy IM. This verifies that the suggested diagnostic technique, utilizing NSC analysis, may detect incipient BRB fault with considerable sensitivity. The escalating trend of NSC with load and speed further substantiates the method resilience.

### 4.2. Fault Detection Accuarcy Analyis and Sensitivity Evaluation

**Figure 9:**
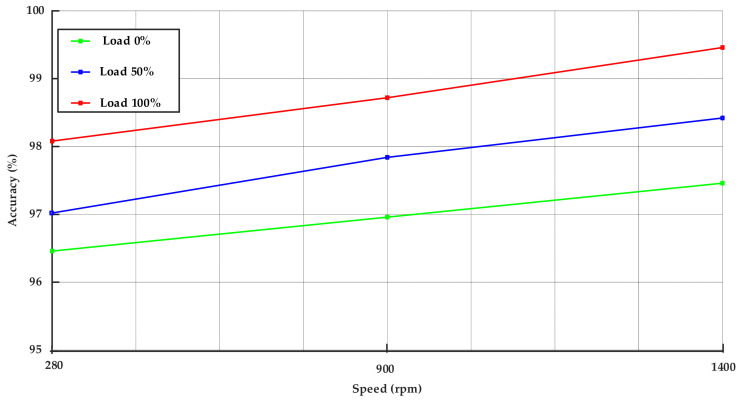
Fault detection accuracy of the proposed method under varying operational conditions.

The fault identification accuracy illustrated in Figure 9 was assessed by comparing the detection outcomes derived from the ST-HAEKF method with the actual IM status (healthy or BRB fault). A detection was deemed successful when the extracted *I*_2*SB*_ component surpassed the threshold *I*_2*H*_.The accuracy for each test condition was computed as the ratio of successfully identified cases to the total number of tests, given as a percentage.

The method consistently shows higher accuracy values, more than 96.5%, especially under full load and higher frequencies, highlighting their superior fault detection performance for the BRB fault.

The impact of inverter-switching frequency on harmonic separation was experimentally examined by evaluating the proposed ST-HAEKF method at switching frequencies 3 kHz, 6 kHz, and 10 kHz, as provided in Table 4. The findings indicated that alterations in switching frequency affect the distribution of high-frequency components in the NSC, although had negligible influence on the estimation of fundamental and fault-related sidebands. The robustness is attributed to the ST-HAEKF execution of time-domain state estimation through innovation-based adaptation, which dynamically modifies the process and measurement noise covariances to address variations in inverter-induced noise. Under the examined conditions, the proposed ST-HAEKF ensures consistent harmonic separation and detection effectiveness across varying inverter-switching frequencies.

Figure 9 demonstrates the accuracy of fault detection for the proposed method across different speed and load conditions. The findings indicate a consistent increase in detection accuracy with higher load and frequency. At 0% load, accuracy varies from approximately 96.5% at a speed of 280 rpm to 97.2% at 1400 Hz. In contrast, at 50% load, accuracy increases from 97% to 98.3% within the same speed range. Maximum performance occurs at full load, with accuracy increasing from 98.1% at 280 rpm to approximately 99.4% at 1400 rpm. This trend arises as increased load levels amplify fault-related current components and enhance the accuracy, whereas elevated supply frequencies facilitate improved spectral separation of sideband components. The proposed approach consistently achieves an accuracy exceeding 96.4% across all operating conditions, thereby demonstrating its robustness and reliability for detecting the BRB fault.

Sensitivity metrics were computed using known fault conditions and detection outcomes. NSC signal-based method demonstrated an average sensitivity of 97.3%, with peak sensitivity at 99.6% under 50–100% load levels. However, at 0–50% load, sensitivity declined to 96.5%.

The comparative results in Table 5 unequivocally indicate that the proposed method consistently surpasses existing approaches across all assessed criteria, attributable to its robust signal representation, load- and speed-invariant feature extraction, and experimentally proven fault sensitivity. The method attains high accuracy and maintains weak fault signatures in noisy environments by concurrently monitoring fundamental components, sidebands, and eliminating baseline noise, surpassing wavelet- and EMD-based techniques that are susceptible to parameter selection. The robust performance under no-load and low-speed situations demonstrates that the extracted features are independent of the operating point, facilitating early problem detection despite variations in torque and speed. In contrast to CNN-based fusion models or machine learning classifiers that depend significantly on the distribution of training data, the suggested method demonstrates robust generalization across high-load, low-load, high-speed, and low-speed conditions, hence ensuring dependable detection in practical applications. The experimental validation across various operating circumstances further substantiates its resilience to disturbances, including sensor noise, mechanical imbalance, establishing it as an efficient and pragmatic option for predictive maintenance in industrial systems.

The proposed ST-HAEKF shows a computational complexity of *O*(*n*^3^ + *m*^3^) per update, where *n* and *m* are the dimension values of the state and measurement vectors, respectively. This study, with *n* = 7 and *m* = 3, has a moderate computational requirement. Real-time implementation tests at a 10 kHz sampling rate indicated an average computation duration of approximately 25 µs per iteration on a standard industrial processor (Intel Core i5, 16 GB RAM, 2.4 GHz), confirming that the method adequately meets the timeliness criteria for online fault detection and monitoring applications. The supplementary adaptable characteristics of the ST-HAEKF impose negligible overhead, rendering the method appropriate for real-time implementation on embedded systems in industrial settings.

### 4.3. Key Contributions of the Proposed Method

We recognize the contributions of previous studies, especially those employing Hilbert spectrum analysis and machine learning methods for the identification of incipient BRB fault. These works have substantially enhanced the domain regarding classification accuracy and feature extraction.

However, our proposed approach has some unique and innovative advantages that outperform existing frameworks in terms of diagnostic reliability particularly under inverter-fed and variable-load conditions in practical application, as detailed below:

1. In contrast to Hilbert- or wavelet-based decomposition methods that passively extract fault-related frequencies, the proposed method continuously tracks the NSC components via a state-space model. The ST-HAEKF concurrently estimates the fundamental and sideband NSC components while adjusting to load-dependent slip fluctuations in real time. This spectrum-tracking capacity guarantees that the sideband frequencies linked to BRBs remain accurately aligned despite variations in speed and load, which are unattainable with fixed-frame Hilbert or Fourier methods.
2. A significant practical constraint of Hilbert spectrum and time-frequency analysis is their susceptibility to inverter harmonics in inverter-driven IMs. Our proposed framework directly represents these harmonics as unmodeled dynamics in the covariance adaption process of the Kalman filter. Consequently, the harmonics associated with the inverter are statistically mitigated, resulting in a more refined and fault-centric NSC representation. This is the initial NSC-based diagnostic system that exhibits harmonic-resilient estimate in inverter-fed drives via adaptive Kalman filtering, to the greatest of our knowledge.
3. While several present investigations depend exclusively on amplitude thresholds or classification methods, our approach incorporates a baseline subtraction technique. This baseline-referenced comparison obviously improves sensitivity to early-stage BRB defects by offsetting intrinsic machine asymmetries and supply discrepancies. The method attains a consistent detection accuracy exceeding 96.5%, even in no-load and low-speed scenarios, where other existing techniques frequently suffer from diminished fault signs.
4. The ST-HAEKF necessitates merely a limited number of recursive state changes, eliminating the requirement for massive training datasets or elaborate feature extraction processes commonly found in intelligent classifiers. Consequently, it is ideally suited for real-time deployment in condition-monitoring systems.

To conclude, our contribution encompasses not only the application of an NSC-based technique but also the development of a reliable, real-time based methodology that identifies diagnostically significant components, adjusts to changes in operating conditions, and removes the necessity of training or classifier adjustment.

## 5. Conclusions

The integration of the Kalman filter-based NSC signal extraction method allows for more effective isolation of fault-related components, significantly improving detection accuracy and reducing noise interference. The NSC method demonstrated high sensitivity, achieving detection rates close to 100% under full load and maintaining rates above 96.5% across all operating conditions, including no-load.

This study demonstrates the effectiveness of the proposed Kalman filter-based NSC extraction method for the reliable detection of the BRB fault in inverter-fed IMs. The findings indicate that NSC signals consistently exhibit higher accuracy values and enhanced sensitivity across various load and speed conditions, establishing them as a more effective, precise, and noise-resistant diagnostic tool. The proposed approach enhances detection accuracy by effectively isolating fault-related components, while also ensuring robustness against inverter-induced harmonics and unavoidable motor asymmetries. The method is valid across both low- and high-slip conditions, ensuring reliable fault visibility independent of load factors.

Future research will concentrate on the development of multi-signal data fusion techniques that integrate NSC with complementary signals, including vibration. This integration may enhance diagnostic reliability by leveraging the strengths of various sensing domains, facilitating early and precise detection of the ITSC and BRB faults in the dynamic conditions of inverter-fed motors.

## Figures and Tables

**Table 1:** Parameters of main equipment for experimental setup.

Parameter	Symbol	Typical Value	Unit
Induction motor	YS-7124	-	-
Motor rated power	P	0.37	kW
Rated rotor speed	n_r_	1400	rpm
Rated current	I_r_	1.12	A
Voltage	U	380	V
Stator resistance	R_s_	0.5	Ohm
Rotor resistance	R_r_	0.3	Ohm
Stator inductance	L_s_	25	mH
Rotor inductance	L_r_	20	mH
Mutual inductance (stator–rotor)	L_m_	15	mH
Number of rotor bars	N_bars_	18	-

**Table 2:** Summary of the NSC amplitude for both healthy IM and faulty IM with one BRB under varying operational conditions.

Operational Conditions		Amplitude of the NSC [A]		Threshold
Speed	Load	Healthy (*I*_2*H*_)	Faulty (*I*_2*SB*_)	
Low	0%	0.029	0.11	*I*_2*SB*_ > *I*_2*H*_
	50%	0.034	0.16	
	100%	0.04	0.21	
High	0%	0.032	0.13	
	50%	0.037	0.37	
	100%	0.056	0.46	

**Table 3:** Simulation results of the NSC amplitude for faulty IM with partial BRB under various operational conditions.

Operational Conditions		Amplitude of the NSC [A]		Fault Severity
Speed	Load	Healthy *I*_2*H*_ by Experiment	Faulty (*I*_2*SB*_) by Simulation	
Low	0%	0.029	0.06	*δ* = 0.25
	50%	0.034	0.07	
	100%	0.04	0.09	
High	0%	0.032	0.08	
	50%	0.037	0.10	
	100%	0.056	0.14	

**Table 4:** The NSC amplitudes at different inverter-switching frequencies under various operational conditions.

Operational Conditions		Amplitude of the NSC (*I*_2*SB*_) [A] at Switching Frequency		
Speed [rpm]	Load [%]	3 kHz	6 kHz	10 kHz
280	0	0.113	0.11	0.106
	50	0.168	0.16	0.158
	100	0.216	0.21	0.203
1400	0	0.134	0.13	0.127
	50	0.375	0.37	0.362
	100	0.467	0.46	0.453

**Table 5:** Comparison of proposed method with different fault detection methods.

Ref.	Method	Sensitivity	High Load	Low Load	High Speed	Low Speed
[6]	Multi-signal fusion with merged convolutional neural network	High	Strong	Good (multi-signal helps)	Strong	Moderate (low speed performance depends on training data)
[11]	Improved empirical mode decomposition for current signals	High	Reliable	Good (EMD extracts weak low load features)	Reliable	Moderate (better than FFT at low speed but still limited)
[18]	Wavelet packet + Fourier features + MLP classifier	High	Good	Limited	Reliable	Weak (MLP needs strong features)
[19]	Focused on inverter-fed IM with imbalanced data	High for incipient faults	Good	Good	Reliable	Reliable
[17]	Sparse Stacked Autoencoder (SSAE) + Light-GBM	High	High	Good	Good	Good
[20]	Demodulated current signal using higher-order energy operator	High (sensitive to incipient faults)	High	Reliable	Reliable	Reliable (better than FFT/HT at low speed)
[28]	Random forest with flux/current features	High	Reliable	Limited	Reliable	Weak (incipient faults masked)
**Proposed method**		High (validated experimentally)	High	High (validated with no load)	High	High (validated at low speed)

## Data Availability

Data is contained within the article.
